# MICU proteins facilitate Ca^2+^-dependent mitochondrial metabolon formation to regulate cellular energetics - independent of MCU

**DOI:** 10.21203/rs.3.rs-6346822/v1

**Published:** 2025-06-26

**Authors:** Henry M. Cohen, Benjamin Gottschalk, Carmen Choya-Foces, Adam Chathoff, Anya Wilkinson, Joanne F. Garbincius, Adyson Johnson, Tyler L. Stevens, Jordan E. Howe, Emily Megill, Jennyfer Ngo, Dhanendra Tomar, Nathaniel W. Snyder, Wolfgang F. Graier, John W. Elrod

**Affiliations:** 1 Aging + Cardiovascular Discovery Center, Lewis Katz School of Medicine at Temple University, Philadelphia, PA; 2 Gottfried Schatz Research Center, Molecular Biology and Biochemistry, Medical University of Graz, Graz, Austria; 3 Department of Internal Medicine, Division of Cardiology, Wake Forest School of Medicine, Winston Salem, NC

## Abstract

Mitochondrial matrix Ca^2+^ concentration ([_matrix_Ca^2+^]) is theorized to be an essential regulator of mitochondrial metabolism by positively regulating key mitochondrial dehydrogenases. However, ablation or functional inhibition of the mitochondrial calcium uniporter channel (mtCU) fails to significantly perturb basal metabolism and is largely phenotypically silent in the absence of stress. This begs the question, what are the primary molecular mechanisms regulating calcium-dependent changes in metabolism? The primary function of MICU proteins (MICU1, MICU2, and MICU3) is reported to be gatekeeping of the mtCU and regulating mitochondrial Ca^2+^ uptake. Here, we demonstrate that MICU proteins function in coordination to impart Ca^2+-^dependent regulation to FADH_2_-dependent mitochondrial dehydrogenases through metabolon formation independent of the mtCU and [_matrix_Ca^2+^]. Our results demonstrate that MICU proteins differentially localize to mitochondrial microdomains and form heterodimers and interactomes in response to intermembrane space Ca^2+^ binding their respective EF-hand domains. Utilizing an equimolar expression platform coupled with unbiased proteomics we reveal unique interactomes for MICU1/2 versus MICU1/3 heterodimers and demonstrate that MICU proteins control coupling of Mitochondrial Glycerol-3-Phosphate Dehydrogenase with Succinate Dehydrogenase/Complex II and impart Ca^2+-^dependent changes in activity. We propose that MICU-mediated mitochondrial metabolons are a fundamental system facilitating matching of mitochondrial energy production with cellular demand and is the primary physiological Ca^2+^ signaling mechanism regulating homeostatic energetics – not mtCU-dependent changes in [_matrix_Ca^2+^].

## Introduction:

Mitochondrial matrix Ca^2+^ concentration ([_matrix_Ca^2+^]) is theorized to be a principal regulator of tricarboxylic acid (TCA) cycle flux by positively regulating the activity of key dehydrogenases located in the mitochondrial matrix. This model is supported by literature dating back over a half-century containing reductionistic and often indirect evidence that [_matrix_Ca^2+^] enhances the activity of key metabolic enzymes that exist within the mitochondrial matrix. The most well characterized calcium control point of metabolism is pyruvate dehydrogenase (PDH)^1^, a multiprotein complex found in the mitochondrial matrix. PDH phosphorylation was first described in 1969 as a key PDH regulatory mechanism governing carbon entry into the TCA cycle^2^. Two years later, the ability of Ca^2+^ to enhance the activity of PDH phosphatase, thereby activating PDH, was described in isolated mitochondria^3^ and supported by isolated enzyme studies^4^. Shortly thereafter in 1979, James McCormack and Richard Denton published a study probing other TCA cycle enzymes for calcium sensitivity, essentially looking for additional Ca^2+^ control points besides PDH. Although the behavior of certain TCA cycle enzymes was altered in high vs. low Ca^2+^ conditions, none of the tested enzymes demonstrated an increase in V_max_ as seen with PDH^5^.

Until recently, the ability to causally interrogate the relationship between [_matrix_Ca^2+^] and mitochondrial energetics was limited by the lack of available tools due to the unknown genetic identity of the transporters responsible for _matrix_Ca^2+^ uptake and efflux. The genetic identity of proteins comprising the mitochondrial calcium uniporter (mtCU), the primary _matrix_Ca^2+^ uptake pathway, were characterized in the 2010’s, with the focus on MCU^6,7^, the mtCU’s essential pore-forming subunit. Additional mtCU components characterized include MICU1^8^, MICU2^9^, MICU3^9^, EMRE^10^, MCUB^11^, and MCUR1^12^. The genetic identity of the mitochondrial sodium/calcium exchanger (NCLX), the primary _matrix_Ca^2+^ efflux pathway, was reported in 2010^13^ and *in vivo* confirmation was provided in 2017^14^. Since these discoveries, several studies have manipulated the mitochondrial calcium handling machinery in numerous model systems with varying results.

Deletion of NCLX in cardiomyocytes in adult mice is acutely lethal^14^. Interestingly, ablation of mtCU function through genetic deletion of *Mcu* or *Emre* is largely phenotypically silent in cardiac and skeletal myocytes^15–19^, brown adipocytes^20^, neurons^21,22^, and other tissues^23^ in the absence of stress. Further, many of these studies demonstrate no detectable metabolic changes with functional ablation of mtCU in the absence of stress^15,17,19^. Upon introduction of pathological stress, ablation of mtCU function through deletion of *Mcu*^15,17^ or *Emre*^19^ or transgenic overexpression of the uniporter’s dominant negative subunit, MCUB^24^ is protective in contexts such as ischemia-reperfusion injury, β-adrenergic stress^15,17,25^, diabetic neuropathy^26^, and Alzheimer’s disease^22^. Even so, the absence of a gross baseline phenotype or substantial metabolic perturbation with ablation of mtCU function is perplexing given the dogmatic importance of matrix calcium signaling in the regulation of metabolism and cellular energetics. Indeed, the concept that Ca^2+^ is the second messenger communicating cellular workload to mitochondria to meet energetic demands is cemented in biology^27,28^.

MICU1 is thought to be the primary regulator of mtCU-mediated _matrix_Ca^2+^ uptake. Current models posit that along with homologs MICU2 and MICU3, MICU1 serves as a rheostat for mtCU-mediated _matrix_Ca^2+^ uptake, tuning the rate of Ca^2+^ uptake relative to intracellular calcium concentrations ([_i_Ca^2+^]) in the microenvironment. According to existing models, MICU2 and MICU3 bind the mtCU in a MICU1-dependent manner. The MICU dimer species (MICU1/1, MICU1/2, or MICU1/3) interacting with MCU/EMRE determines the uniporter’s propensity for _matrix_Ca^2+^ uptake relative to local inner-membrane space (IMS) [Ca^2+^]^29^. Although certain aspects of MICU-mediated regulation are debated, such as if MICU1 occludes the mtCU basally or potentiates uptake^30–35^, multiple groups have shown energetic consequence to MICU deletion^8,32,36–38^ which were rationalized by altered mtCU regulation causing a change in [_matrix_Ca^2+^] and subsequently altering mitochondrial dehydrogenase activity^39^. Unlike deletion of MCU, MICU1 deletion does not ablate mtCU functionality and is embryonic lethal in both drosophila^40^ and in mice^41^. Existing models would explain this embryonic lethality by _matrix_Ca^2+^ overload caused by ungated mtCU-mediated _matrix_Ca^2+^ uptake resulting in metabolic derangement and fulminant cell death. However, the lethality caused by *Micu1* deletion is not rescued by ablating mtCU function through deletion of MCU (*Mcu*^−/−^ × *Micu1*^−/−^) ^40^ or EMRE (*Emre*^−/−^ × *Micu1*^−/−^) ^41^ and neither EMRE (*Emre*^−/−^) nor MCU (*Mcu*^−/−^) deletion is lethal, suggesting MICU1 functions independently of its role in regulating the uniporter. In support, we recently discovered that MICU1 regulates mitochondrial architecture independent of the uniporter ^42^. This work is corroborated by a previous study demonstrating MICU1 modulates cristae junction integrity and spatially anchors the uniporter to the inner boundary membrane during stimulation^43^.

The discrepancy in phenotypic consequence between MCU deletion and MICU1 deletion suggests that the existing _matrix_Ca^2+^-centric model of mitochondrial calcium signaling is incomplete. Oxidative phosphorylation and ATP production are Ca^2+^ sensitive although the mechanisms of this are debated^44–46^. Thus, we have a significant gap in knowledge; *How does i*^*Ca2*+^ *signaling regulate metabolism if not*_*matrix*_*Ca*^*2+*^ *uptake?*

Here, we demonstrate that MICU1, MICU2, and MICU3 function in coordination to impart Ca^2+^-dependent regulation to key metabolic complexes fundamental to mitochondrial metabolism independent of the mtCU and [_matrix_Ca^2+^]. Our results suggest that MICU proteins are differentially localized to mitochondrial microdomains and that MICU heterodimer formation occurs upon Ca^2+^ binding their respective EF-hand domains in the IMS. In support, physiological signaling, such as IP_3_R-Ca^2+^ release, elicits a near-instant increase in MICU1/2 heterodimerization at the inner boundary membrane. Utilizing an equimolar expression system coupled with unbiased proteomics we reveal unique interactomes for MICU1/2 versus MICU1/3 heterodimers. Highlighting the physiological importance of this system, we identified that MICU1/2 heterodimers interact with mitochondrial Glycerol-3-phosphate dehydrogenase (GPD2) and Succinate Dehydrogenase/Complex II (C-II) to couple TCA cycle metabolite flux and oxidative phosphorylation. We propose that MICU dimer formation and sub-mitochondrial localization is a fundamental system to facilitate [_IMS_Ca^2+^]-sensitive metabolon formation and localization. Our results suggest this may be the primary physiological Ca^2+^ signaling mechanism regulating cellular energetics.

## Results:

### MICU proteins are in high-molecular weight protein complexes lacking the mitochondrial calcium uniporter

**Figure 1.**
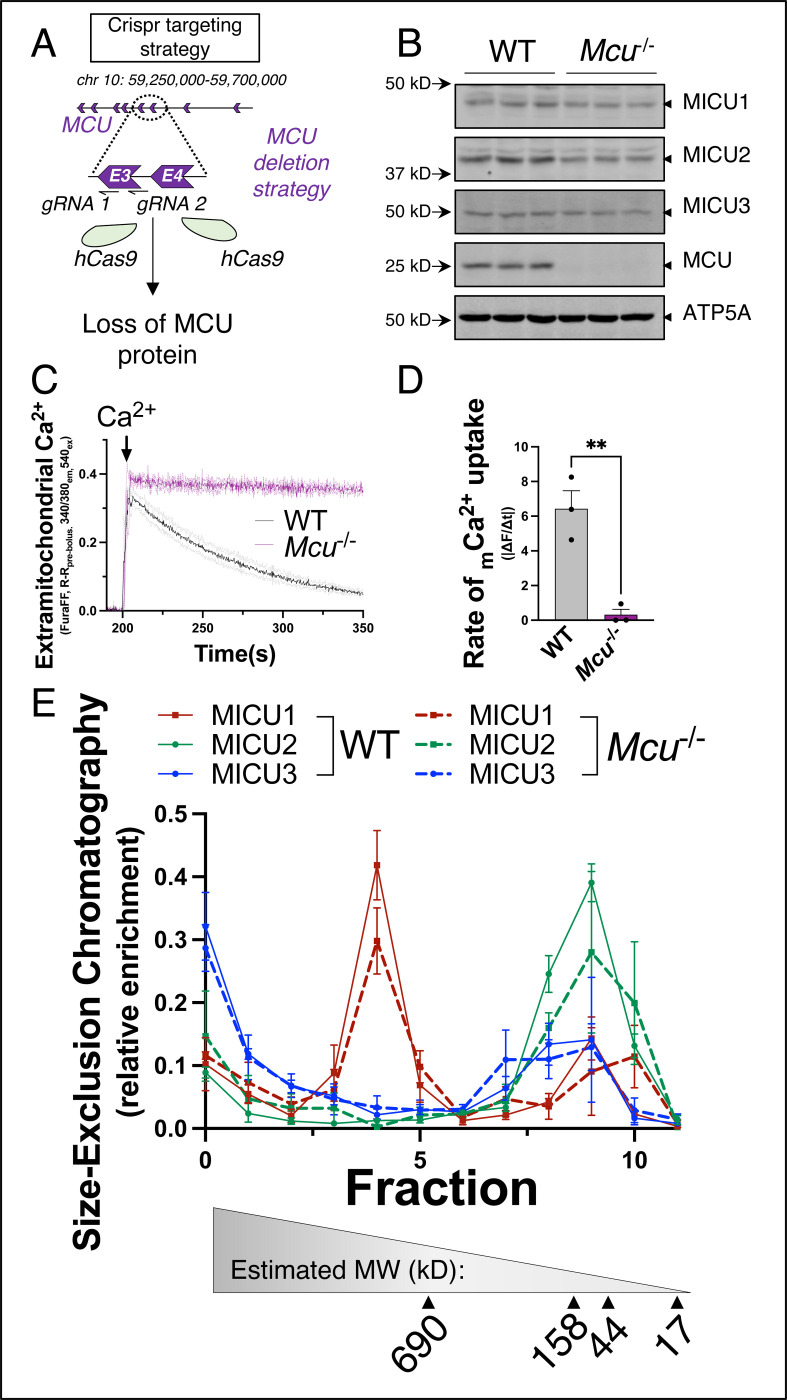
MICU proteins are in high-MW protein complexes lacking the uniporter. **A.** Schematic of *Mcu* gene targeting strategy for generation of *Mcu*^−/−^ Neuro-2a (N2a) cells. **B.** Western blot analysis of MICU1, MICU2, MICU3, MCU, and ATP5a (loading control) in *Mcu*^−/−^ N2a cells. **C, D.** Recordings of extramitochondrial calcium in response to a 5 μM Ca^2+^ bolus in *Mcu*^−/−^ and control N2a cells. Data presented as |(mean ± SEM)*10^3^|. Unpaired t-test. **=p<0.01. **E.** Quantification of size exclusion chromatography protein fractionation samples performed on WT and *Mcu*^*−/−*^ N2a cells. Data presented as mean ± SEM. Two-way Anova with Tukey’s multiple comparison test.

Utilizing CRISPR/Cas9n, we generated *Mcu*^−/−^ Neuro-2a (N2a) cells (Figure 1A-B) to determine if acute _matrix_Ca^2+^ uptake is required for protein stability of MICU1, MICU2, or MICU3. Interestingly, MICU1, MICU2, and MICU3 are stable in the context of *Mcu* deletion and ablation of acute _matrix_Ca^2+^ uptake (Figure 1C-D), suggesting expression and post-translational stability is independent of the uniporter. To further investigate the MICU proteomes, lysates were collected from *Mcu*^*−/−*^ and wild-type (WT) N2a cells and subjected to size-exclusion chromatography under non-reducing conditions. Similar to our previously published results^42^, the fractional distribution of MICU1, MICU2, and MICU3 is unaffected by deletion of *Mcu* (Figure 1E), demonstrating that MICU proteins are retained in high-molecular weight (MW) complexes independent of binding the uniporter and acute _matrix_Ca^2+^ uptake. Altogether, these results support that the functional relevance of MICU proteins is independent of the mtCU and _matrix_Ca^2+^ uptake and that their post-translational stability (expression) is interdependent.

### MICU dimerization and stoichiometry are independent of the uniporter and acute _matrix_Ca^2+^ uptake

**Figure 2.**
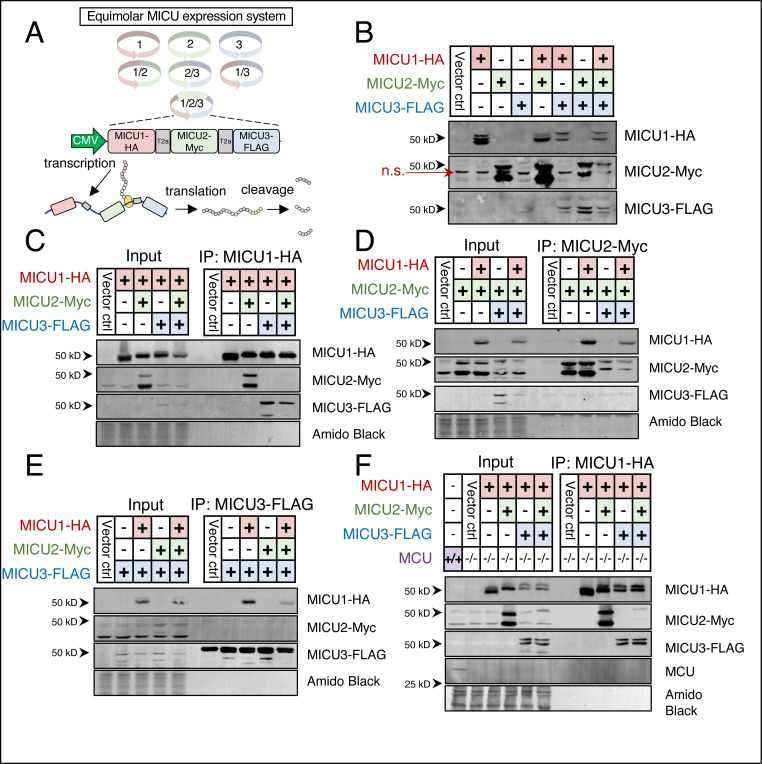
MICU dimerization and stoichiometry are independent of the uniporter and acute _matrix_Ca^2+^ uptake. **A.** Schematic of MICU1, MICU2, and MICU3 equimolar expression system. **B.** Western blot demonstrating functional validation of constructs. n.s. indicates nonspecific bands. Also note that open reading frames are ordered MICU1→ MICU2→ MICU3 on all dual/triple expression constructs and 17 of the 18 amino acids of the T2a linker remains on the upstream ORF following cleavage, explaining the slight shift seen on HA, Myc, and FLAG blots in dual and triple expression constructs. **C.** Immunoprecipitation (IP) of MICU1-HA from *Micu1*^−/−^ HEK 293T cells expressing MICU1-HA, MICU1-HA-T2a-MICU2-Myc, MICU1-HA-T2a-MICU3-FLAG, and MICU1-HA-T2a-MICU2-Myc-T2a-MICU3-FLAG. IP products used for western blots of HA, Myc, and FLAG. **D.** IP of MICU2-Myc from *Micu1*^−/−^ HEK 293T cells expressing MICU2-Myc, MICU1-HA-T2a-MICU2-Myc, MICU2-Myc-T2a-MICU3-FLAG, and MICU1-HA-T2a-MICU2-Myc-T2a-MICU3-FLAG. **E.** IP of MICU3-FLAG from *Micu1*^−/−^ HEK 293T cells expressing MICU3-FLAG, MICU1-HA-T2a-MICU3-FLAG, MICU2-Myc-T2a-MICU3-FLAG, and MICU1-HA-T2a-MICU2-Myc-T2a-MICU3-FLAG. **F.** Immunoprecipitation (IP) of MICU1-HA from *Mcu*^−/−^ HEK 293T cells expressing MICU1-HA, MICU1-HA-T2a-MICU2-Myc, MICU1-HA-T2a-MICU3-FLAG, and MICU1-HA-T2a-MICU2-Myc-T2a-MICU3-FLAG.

Dimeric MICU proteins are thought to be a primary regulator of mtCU open probability. The stoichiometry of the MICU homo- or heterodimer tethered to the mtCU (MICU1/1, MICU1/2, or MICU1/3) is thought to confer unique properties to modulate the dynamics of _matrix_Ca^2+^ uptake^9,31^. Further, it has been suggested that MICU dimer formation occurs only after MICU1 has bound MCU/EMRE and that the mtCU itself serves as the platform for MICU dimerization^47^. To test if MICU proteins can dimerize in the absence of mtCU, we generated a system to express all possible permutations of MICU proteins (MICU1, MICU2, and MICU3) in equimolar ratios. Constructs were created with MICU open reading frames separated by a t2a linker peptide and expressed under the control of a single cytomegalovirus (CMV) ubiquitous promoter such that, following spontaneous post-translational cleavage of the t2a linker, expression level was equal for all MICUs (Figure 2A-B). MICU1-HA was expressed alone, with MICU2 (MICU1-HA-t2a-MICU2-Myc), with MICU3 (MICU1-HA-t2a-MICU3-FLAG), or with both MICU2 and MICU3 (MICU1-HA-t2a-MICU2-Myc-t2a-MICU3-FLAG) in *Micu1*^*−/−*^ and *Mcu*^*−/−*^ cells (Figure 2C, 2F). MICU1-HA was immunoprecipitated (IP’d) using the C-terminal HA epitope tag and probed for MICU2-Myc and MICU3-FLAG. MICU2 and MICU3 both co-immunoprecipitated with MICU1 when co-expressed exclusively with MICU1 in both *Micu1*^*−/−*^ and *Mcu*^*−/−*^ HEK293T cells. When MICU2-Myc was expressed alone or with MICU1-HA and/or MICU3-FLAG (MICU2-Myc, MICU1-HA-t2a-MICU2-Myc, MICU2-Myc-t2a-MICU3-FLAG, MICU1-HA-t2a-MICU2-Myc-t2a-MICU3-FLAG) and IP’d using the C-terminal Myc-tag, we saw that MICU1-HA co-IP’d with MICU2-Myc whenever present, however MICU3-FLAG failed to co-IP with MICU2-Myc (Figure 2D). Similarly, when MICU3-FLAG was expressed alone or with MICU1-HA and/or MICU2-Myc (MICU3-FLAG, MICU1-HA-t2a-MICU3-FLAG, MICU2-Myc-t2a-MICU3-FLAG, MICU1-HA-t2a-MICU2-Myc-t2a-MICU3-FLAG) and IP’d using the C-terminal FLAG-tag we found that MICU1-HA co- IP’s with MICU3-FLAG whenever present, however MICU2-Myc does not co-IP with MICU3-FLAG (Figure 2E). Taken together, these data demonstrate that MICU2 and MICU3 do not heterodimerize and that MICU dimerization and stoichiometry are independent of the uniporter and acute _matrix_Ca^2+^ uptake. Further, these data demonstrate that specific molecular systems besides transcript or peptide abundance govern MICU heterodimer formation.

### MICU heterodimer formation is sensitive to [_IMS_Ca^2+^]

**Figure 3.**
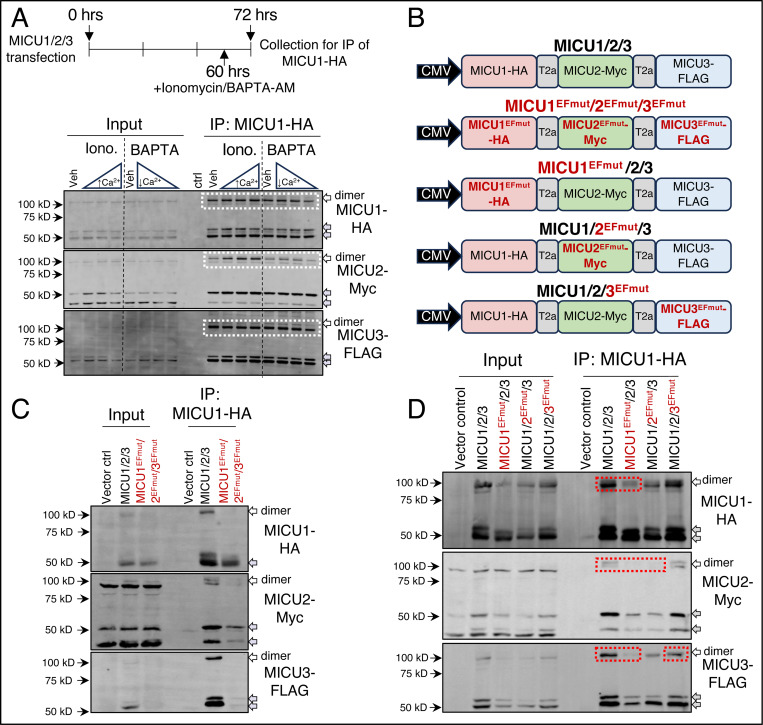
MICU heterodimer formation is sensitive to [_IMS_Ca^2+^]. **A.** Immunoprecipitation (IP) of MICU1-HA from HEK 293T cells expressing MICU1-HA-T2a-MICU2-Myc-T2a-MICU3-FLAG treated with Ionomycin (Veh, 1.5, 3, or 6 μM) or BAPTA-AM (Veh, 2.5, 5, or 10 μM) 12 hours prior to collection for IP. Western blots performed on IP products for HA, Myc, and FLAG. Monomeric protein species are indicated by grey arrow and dimeric protein species are indicated by white arrows with black outline. **B.** Schematic of equimolar MICU protein expression system harboring MICU1^EFmut^ (D237A, E248K, D427A, E438K), MICU2^EFmut^ (D185A, E196K, D375A, E386K), and/or MICU3^EFmut^(D245A, E256K, D483A, E494K). **C.** IP of MICU1-HA from WT HEK 293T cells expressing MICU1-HA-T2a-MICU2-Myc-T2a-MICU3-FLAG or MICU1^EFmut^-HA-T2a-MICU2^EFmut^-Myc-T2a-MICU3^EFmut^-FLAG. Western blots performed on IP products for HA, Myc, and FLAG. **D.** IP of MICU1-HA from WT HEK 293T cells expressing MICU1-HA-T2a-MICU2-Myc-T2a-MICU3-FLAG, MICU1^EFmut^-HA-T2a-MICU2-Myc-T2a-MICU3-FLAG, MICU1-HA-T2a-MICU2^EFmut^-Myc-T2a-MICU3-FLAG, or MICU1-HA-T2a-MICU2-Myc-T2a-MICU3^EFmut^-FLAG. Monomeric protein species are indicated by grey arrow and dimeric protein species are indicated by white arrows with black outline.

We next designed experiments to define the mechanisms governing MICU dimerization. MICU1, MICU2, and MICU3 each contain two conserved EF-hand domains essential for their ability to sense environmental [Ca^2+^]. Previous isolated protein studies have suggested that MICU dimer formation is calcium sensitive^47,48^; to our knowledge this has not been shown in an *in vitro* or *in vivo* system. Therefore, we hypothesized that local [_IMS_Ca^2+^] controls MICU dimer formation. To test this, cells transfected with MICU1-HA-T2a-MICU2-Myc-T2a-MICU3-FLAG were treated with the Ca^2+^ ionophore, ionomycin (Veh, 1.5, 3, 6 μM), or the cell-permeable Ca^2+^ chelator, BAPTA-AM (Veh, 2.5, 5, 10μM), 12h prior to lysate collection. IP of MICU1-HA revealed an increase in MICU1/2 heterodimer formation in response to increasing [Ionomycin] and a decrease in MICU1/2 heterodimer formation in response to Ca^2+^-chelation, increasing [BAPTA-AM] (Figure 3A). To examine this further, we inserted EF-hand point mutations in our MICU1, MICU2, and MICU3 equimolar expression system such that all MICUs independently, and in combination, were rendered insensitive to Ca^2+^ (Figure 3B). The triple mutant construct and WT constructs were expressed and IP of MICU1-HA or MICU1^EFmut^-HA were performed. Loss of Ca^2+^-binding significantly reduced MICU heterodimer formation (Figure 3C). To investigate this further, MICU1, MICU2, and MICU3 triple expression plasmids were generated harboring EF-hand mutations of MICU1, MICU2, or MICU3 (MICU1^EFmut^/2/3, MICU1/2^EFmut^/3, MICU1/2/3^EFmut^) and expressed in HEK 293T cells. IP of the C-terminal HA tag (MICU1-HA and MICU1^EFmut^-HA) revealed decreased formation of the MICU1/MICU2 heterodimer upon mutation of EF-hands on either MICU1 or MICU2 (Figure 3D) indicated by loss of the dimer band, further supporting that the MICU1/MICU2 heterodimer forms most readily when bound Ca^2+^. These data demonstrate that Ca^2+^ binding the EF-hand domains of individual MICU proteins dictates dimerization and stability.

### Physiological Ca^2+^ signaling acutely increases MICU1/2 heterodimerization at the mitochondrial inner boundary membrane

**Figure 4.**
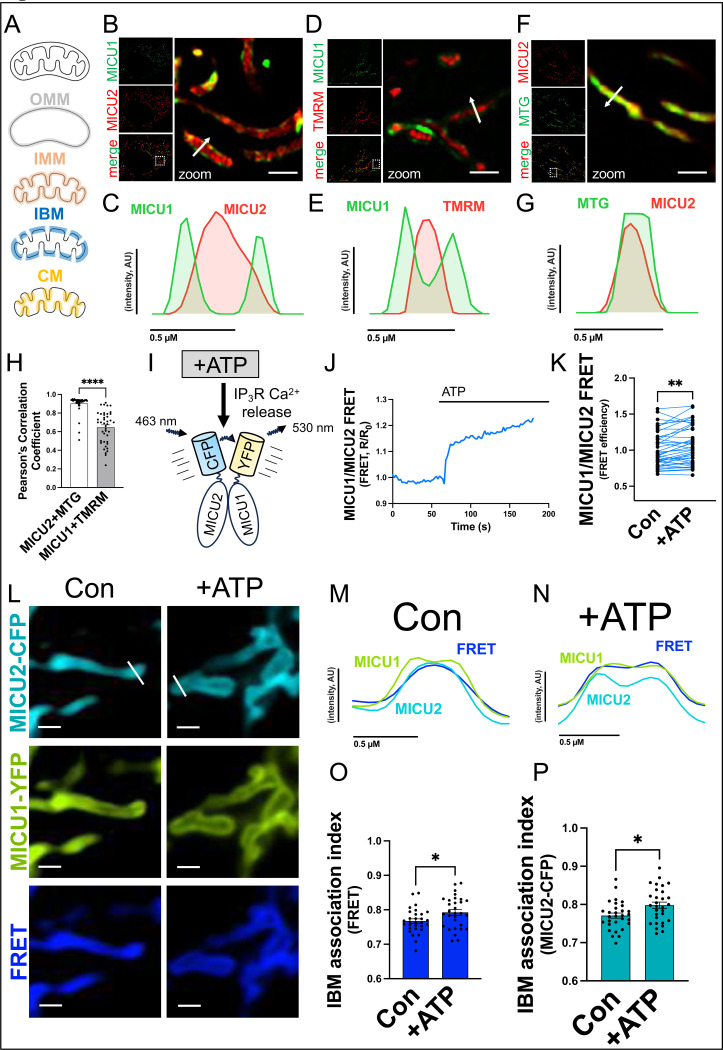
Physiological Ca^2+^ signaling acutely increases MICU1/2 heterodimerization at the mitochondrial inner boundary membrane. **A.** Schematic of whole mitochondria, outer mitochondrial membrane (OMM), inner mitochondrial membrane (IMM), inner boundary membrane (IBM), and cristae membrane (CM). **B, C.** Dual SIM imaging of HeLa cells expressing MICU1-EGFP and MICU2-mCherry and accompanying line profiles. **D, E.** Dual SIM imaging of HeLa cells expressing MICU1-EGFP and stained with mitochondrial matrix marker TMRM and accompanying line profiles. **F, G.** Dual SIM imaging of HeLa cells expressing MICU2-mCherry and stained with mitochondrial matrix marker MitoTracker green (MTG) and accompanying line profiles. **H.** Pearson’s Correlation Coefficient of MICU1-EGFP/TMRM and MICU2-mCherry/MTG. **I.** Schematic of MICU2-CFP/MICU1-YFP Fluorescence Resonance Energy Transfer (FRET) system used to visualize changes in spatial relationships of MICU protein pairs in HeLa cells stimulated with purinergic agonist ATP. **J.** Representative trace of FRET signal generated by HeLa cells expressing MICU2-CFP/MICU1-YFP FRET pair were imaged in buffer containing 2 mM CaCl_2_ and stimulated with ATP. **K.** FRET signal before and after ATP treatment. **L.** Individual mitochondria in HeLa cells expressing MICU2-CFP/MICU1-YFP FRET pair before and after ATP treatment. **M, N.** line profiles of cells before and after ATP treatment **O, P.** Inner boundary membrane association index of FRET signal and MICU2-CFP before and after ATP treatment. Unpaired t-test. ****p<0.0001 **p<0.01 *p<0.05. Scale bars are ~1 μM.

Our co-authors have previously shown that MICU1 localizes to the inner boundary membrane^43^. Given the [_IMS_Ca^2+^]-responsiveness of MICU1/2 heterodimer formation, we hypothesized that monomeric MICU1, monomeric MICU2, and the MICU1/MICU2 heterodimer occupy spatially distinct microdomains within the mitochondria. To address this hypothesis, two channel structured illumination microscopy (dual-SIM) was performed on HeLa cells expressing MICU1-EGFP and MICU2-mCherry (figure 4B-C). Interestingly MICU1 localized to the inner boundary membrane; however, MICU2 localized to the cristae membrane independent of MICU1. This was confirmed by expressing MICU1-EGFP or MICU2-mCherry together with mitochondrial matrix markers TMRM or MitoTracker green, respectively (Figure 4D-H). Next, we tested if the spatial relationship between MICU1 and MICU2 may itself be sensitive to changes in [_IMS_Ca^2+^]. To this end, YFP/CFP FRET probes were designed for MICU1 and MICU2 (MICU1-YFP, MICU2-CFP) to visualize and quantify the physical approximation of MICU1 and MICU2 in live cells and how interactions may change in response to physiological stimulation (Figure 4I). After recording baseline signals (FRET), cells were stimulated with the purinergic receptor agonist, ATP, to elicit IP_3_R-mediated Ca^2+^ release from the endoplasmic reticulum. Upon ATP stimulation, FRET signal generated by the MICU1/2 FRET pair acutely increased (Figure 4J, K). Next, we sought to understand if the increase in MICU1/2 heterodimer formation occurred within a specific microdomain of the mitochondrion. To this end, recordings were acquired of cells expressing the MICU1/2 FRET pair before and after ATP stimulation (Figure 4L-N). Interestingly, the inner boundary membrane association index of both MICU2-CFP and the FRET signal increased in response to ATP (Figure 4O, P). Taken together these data demonstrate that receptor signaling-dependent increases in [_IMS_Ca^2+^] increase MICU1/2 heterodimer formation at the mitochondrial inner boundary membrane.

### MICU heterodimers display differential interactomes

**Figure 5.**
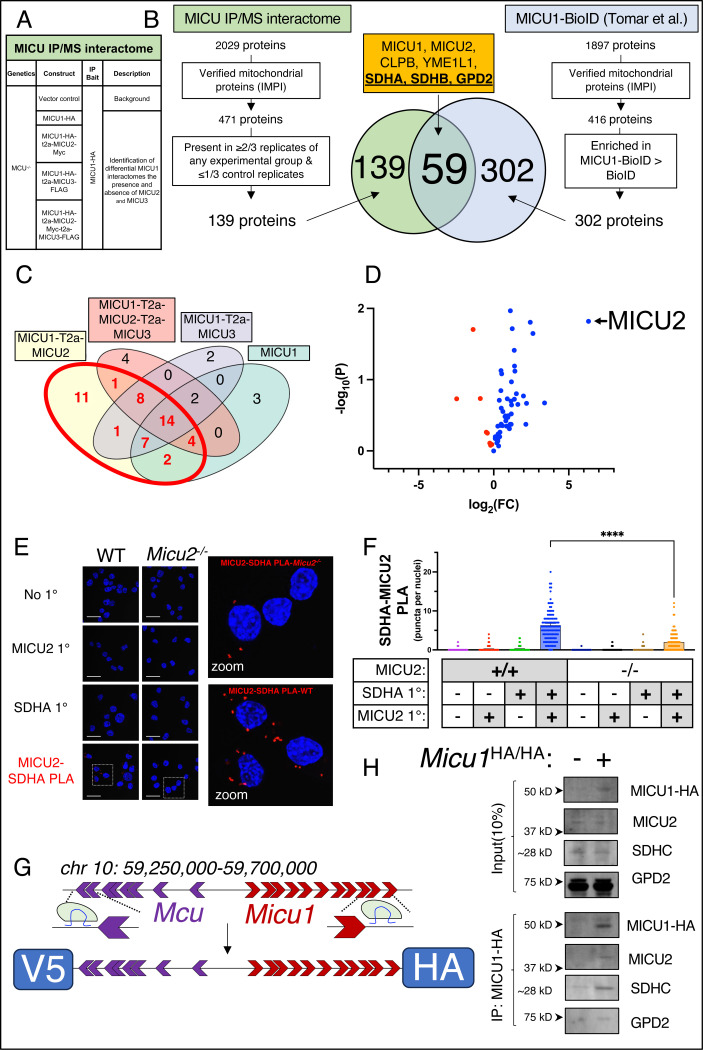
MICU1/MICU2 heterodimers interact with FADH_2_-dependent mitochondrial dehydrogenases. **A.** *Mcu*^−/−^ HEK 293T cells were transfected with control plasmid, MICU1-HA, MICU1-HA-T2a-MICU2-Myc, MICU1-HA -Myc-T2a-MICU3-FLAG, or MICU1-HA-T2a-MICU2-Myc-T2a-MICU3-FLAG and lysates uses for IP of MICU1-HA. IP products were used for IP/LC/MS. **B.** Indicated exclusionary criteria were applied to 2029 peptides identified to generate 139 proteins of interest. The indicated exclusionary criteria were applied to previously published MICU1-BioID^42^ dataset to generate 302 proteins of interest. 59 proteins were found in both the 139 hits from our IP/MS MICU interactome screen and the 302 MICU1-BioID hits. **C.** Venn diagram of 59 putative MICU interactors indicating which experimental group(s) of the MICU IP/MS interactome screen each of the 59 targets were identified in. **D.** Volcano plot of the 59 interactors identified through proteomic screen comparing enrichment in the MICU1-HA-T2a-MICU2-Myc and MICU1-HA-T2a-MICU3-FLAG groups. **E, F.** Proximity ligation assay was performed on *Micu2*^*−/−*^ and WT N2a cells with no 1° antibody and single 1° antibody controls. Scale bars are ~25 μM. **G.** Schematic of MICU1-HA-MCU-V5 knock-in mouse allele. **H.** Co-immunoprecipitation of MICU1-HA, MICU2, SDHC, and GPD2 from hepatic mitochondria isolated from MICU1-HA knock in mouse. Two-way ANOVA with Šídák’s multiple comparison test. ****p<0.0001.

To define the mitochondrial complexes interacting with MICU proteins we evaluated the mtCU-independent interactome of MICU1 in a co-expression system with MICU2 and/or MICU3. All permutations of MICU1-containing plasmids (MICU1-HA, MICU1-HA-T2a-MICU2-Myc, MICU1-HA-T2a-MICU3-FLAG, MICU1-HA-T2a-MICU2-Myc-T2a-MICU3-FLAG) were expressed in *Mcu*^*−/−*^ HEK 293T cells (Figure 5A). IPs of MICU1-HA were performed and prepped for liquid-chromatography mass-spectrometry (LC/MS). Bait protein (MICU1-HA) was identified in all experimental replicates and none of the vector control replicates. 471 of 2209 unique proteins were found in databases of known mitochondrial proteins^49,50^. 139 of these 471 proteins were present in at least 2 of 3 replicates of one experimental group without being identified in more than 1 of 3 replicates of the control group. Next, cross-referenced this dataset with our previously published MICU1-BioID2 proximity biotinylation dataset ^42^. In brief, we expressed a MICU1-BioID2 fusion protein harboring a C-terminal biotin ligase capable of biotinylating peptides <10 nm in distance. Biotinylated proteins were isolated via streptavidin pull-down and identified by LC/MS. 1897 unique proteins were identified through this analysis and only verified mitochondrial proteins that were enriched over BioID2 control were considered, revealing 302 target proteins. 59 proteins were identified in both the MICU1-BioID2 dataset and the MICU IP interactome dataset including MICU1, MICU2, and verified interactors reported in the literature CLPB^51^, and YME1L1^36^ (Figure 5B). 48 of the 59 MICU interactors appeared in at least 2/3 replicates of the MICU1/2 co-expression group with many demonstrating limited enrichment in other experimental groups demonstrating unique interactomes for different MICU dimer species (Figure 5C,D).

Intriguingly, Complex II (C-II)/Succinate Dehydrogenase (SDH) subunits Succinate dehydrogenase flavoprotein subunit A (SDHA) and Succinate dehydrogenase iron-sulfur subunit B (SDHB), as well as Glycerol-3-phosphate dehydrogenase (GPD2) were high-value hits from our proteomics studies (Figure 5B). It has previously been reported that SDH/C-II and GPD2 reside in the same multiprotein complex ^52^ providing strong evidence of MICU1/2 heterodimer interaction with a GPD2/SDH multiplex. Both GDP2 and SDH feed FADH_2_-derived electrons to complex III (C-III) in the form of reduced coenzyme Q (CoQ). Currently there is no known physical or functional relationship between any MICU protein and C-II or GPD2. In addition, calcium control of the electron transport chain has been hotly debated, but this was almost entirely based on [_matrix_Ca^2+^] and mtCU-dependent uptake^44,46,53^.

### MICU1/2 heterodimers interact with succinate dehydrogenase/C-II and glycerol-3-phosphate dehydrogenase

Next, we validated the physical proximity and interaction of the MICU1/2 heterodimer and SDH by performing a proximity ligation assay (PLA) in WT and *Micu2*^*−/−*^ N2a cells using validated MICU2 and SDHA antibodies such that red puncta form only when the target proteins are within 40-nm (Figure 5E). Significantly more puncta formed in WT cells than *Micu2*^*−/−*^ cells, indicating that MICU2 and SDHA are in near proximity (Figure 5F). However, MICU2 is found within the IMS and SDHA is reported to localize to the matrix-facing end of C-II^54^; thus, the inner mitochondrial membrane separating these compartments makes direct physical interaction between MICU2 and SDHA highly unlikely. Therefore, we conjectured that physical interaction is likely occurring by MICU1/2 binding C-II’s transmembrane subunits SDHC and/or SDHD. To this end, we generated a *Micu1*-HA, *Mcu*-V5 double knock-in mouse which harbors an HA- and V5-tag sequence within the endogenous *Micu1* and *Mcu* gene loci, respectively, such that the endogenous proteins harbor unique C-terminal epitope tags (Figure 5G, Supplemental Figure 2). Hepatic mitochondria were isolated from *Micu1*^HA/HA^ and control mice and an MICU1-HA IPs were performed. We chose to isolate mitochondria from hepatocytes since MICU1 and MICU2 are heavily enriched in hepatocytes^9^. IP of MICU1-HA demonstrated that endogenous MICU1/2 heterodimers directly interact with SDHC and GPD2 (Figure 5H). Altogether, these data demonstrate that MICU1/2 heterodimers interact with SDH/C-II and GPD2 at the inner boundary membrane.

### MICU2 confers calcium sensitivity to the SDH/GPD2 metabolon

**Figure 6.**
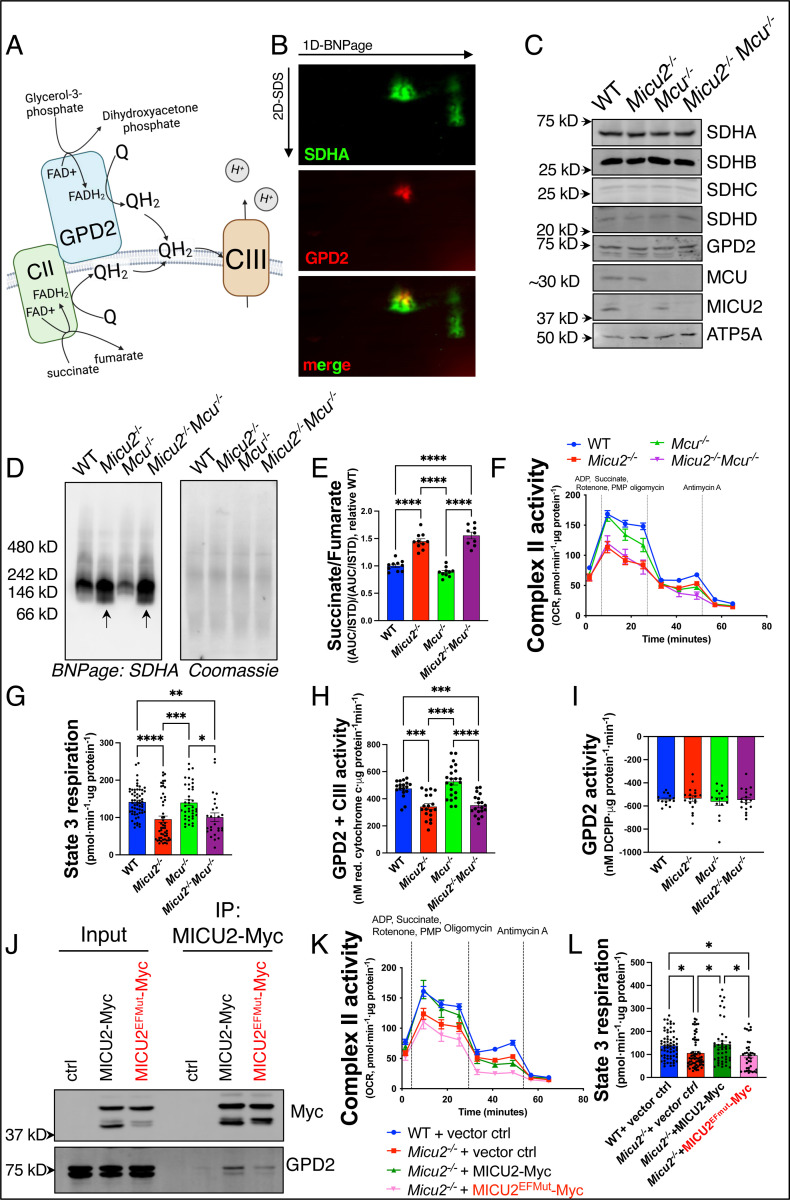
MICU2 confers Ca^2+^ sensitivity to the SDH/GPD2 metabolon. **A.** Schematic of FADH_2_-dependent dehydrogenases SDH/C-II and GPD2 feeding electrons to CIII via QH_2_ **B.** 2D protein gel electrophoresis performed on mitochondria isolated from N2a cells. **C.** Western blot analysis of lysates from WT, *Micu2*^−/−^, *Mcu*^−/−^, and *Micu2*^−/−^*Mcu*^−/−^ N2a cells for SDHA, SDHB, SDHC, SDHD, GPD2, MCU, MICU2, and ATP5a (loading control). **D.** BlueNative-PAGE (BNPage) performed on isolated mitochondria. **E.** Quantification of resting succinate/fumarate ratio using LC/MS. **F, G.** Complex II activity assay oxygen consumption rate (OCR) with accompanying quantification of state 3 respiration. **H.** GPD2+CIII activity measured in isolated mitochondria. **I.** GPD2 activity measured in isolated mitochondria. **J.** Co-IP of MICU2-Myc/MICU2^EFmut^-Myc and GPD2 from HEK 293T cells. **K, L**. Complex II activity assay OCR and accompanying quantification of state 3 respiration. Data presented as mean ± SEM. One-way ANOVA with Tukey’s multiple comparison test. *p<0.05, **p<0.01, ****p<0.0001.

SDH is a heterotetrametric enzyme (subunits: SDHA, SDHB, SDHC, and SDHD) residing within the inner mitochondrial membrane, converting succinate to fumarate and reducing FAD^+^ to FADH_2_ as a component of both the TCA cycle and electron transport chain (ETC). GPD2 converts glycerol-3-phosphate (G3P) to dihydroxyacetone phosphate (DHAP) and, like SDH, feeds electrons to C-III via reduced CoQ (Figure 6A). Given the functional relationship of SDH and GPD2 and physical proximity to MICU1/2, we hypothesized that GPD2 and SDH form a calcium-dependent metabolon for efficient coupling of this defined metabolic process.

To characterize formation of this metabolon and functional coupling of SDH and GPD2 we performed 2D protein gel electrophoresis and found that SDHA and GPD2 are in a common complex (Figure 6B). Next, we sought to understand the functional relationship between MICU2 and the SDH/GPD2 metabolon. To this end, we generated WT, *Micu2*^−/−^, *Mcu*^−/−^, and *Micu2*^−/−^ x *Mcu*^−/−^ N2a cells (Figure 6C) to measure activity/function of the metabolon and to determine if any observed changes are MCU-dependent. Since C-II activity is reported to be regulated at the level of complex assembly^55,56^, we first examined if deletion of MICU2 altered C-II assembly. Mitochondria were isolated from WT, *Micu2*^−/−^, *Mcu*^−/−^, and *Micu2*^−/−^ x *Mcu*^−/−^ cells and blue native page (BN-page) was performed to evaluate C-II assembly in native conditions. BN-page analysis demonstrated a downward shift in an SDHA containing complex in *Micu2*^−/−^ and *Micu2*^−/−^ x *Mcu*^−/−^ cells relative to control and *Mcu*^−/−^ cells (Figure 6D), demonstrating a MICU2-dependent and MCU-independent change in size of the SDH/C-II complex. Next, we sought to understand the functional implications of loss of MICU2 on C-II. Metabolomic analysis of TCA cycle intermediates in WT, *Micu2*^−/−^, *Mcu*^−/−^, and *Micu2*^−/−^ x *Mcu*^−/−^ cells revealed a MICU2-dependent and MCU-independent increase in the succinate/fumarate ratio (Figure 6E), suggestive of decreased oxidation of succinate by C-II with loss of MICU2 that is unaffected by the loss of MCU and acute _matrix_Ca^2+^ uptake. This result was corroborated by a C-II activity assay in which intact cells were permeabilized and provided succinate, ADP, and rotenone to inhibit complex I such that respiration (i.e., rate of oxygen consumption) is directly a result of C-II activity (Figure 6F). *Micu2*^−/−^ and *Micu2*^−/−^ x *Mcu*^−/−^ cells demonstrated significantly decreased state 3 respiration, with no change in state 4 respiration, and a trending decrease in the respiratory control ratio (Figure 6G, Supplemental Figure 3). These data demonstrate that MICU2 regulates C-II activity independent of uniporter and _matrix_Ca^2+^ uptake.

Next, we examined if GPD2 activity was impacted by loss of MICU2. To this end, we performed a GPD2 + C-III activity assay in which we provided G3P to isolated mitochondria treated with rotenone to inhibit complex I (C-I) and measured cytochrome c reduction by C-III, as previously reported^57,58^. *Micu2*^−/−^ and *Micu2*^−/−^ x *Mcu*^−/−^ mitochondria demonstrated a significant reduction in GPD2 + C-III activity (Figure 6H). Interestingly, when isolated mitochondria are 1) fed G3P as a substrate, 2) C-III is inhibited using antimycin A, 3) CI is inhibited with rotenone, and GPD2 activity is directly measured (Figure 6I) there is no difference between genotypes, suggesting that the observed metabolic deficit caused by MICU2 loss is due to altered coupling rather than deficiency of GPD2 itself.

Finally, we examined if MICU2 confers Ca^2+^-sensitivity to the SDH/GPD2 metabolon through differential association of Ca^2+^-free versus Ca^2+^-bound MICU2. To this end, we performed IPs with either MICU2-myc or MICU2^EFmut^-Myc (D185A, E196K, D375A, E386K) expressing cells. Interestingly, the physical interaction between GPD2 and MICU2 was destabilized by mutation of the EF-hands suggesting that Ca^2+^-bound MICU2 more strongly associates with the SDH/GPD2 metabolon. To investigate whether Ca^2+^-bound MICU2 differentially affects function of the SDH/GPD2 metabolon MICU2 and MICU2^EFmut^ were expressed in *Micu2*^*−/−*^ cells. Rescue of MICU2, but not MICU2^EFmut^, was sufficient to restore C-II activity (Figure 6K,L). Taken together these results demonstrate that MICU2 is necessary for coupling of the SDH/GPD2 metabolon to oxidative phosphorylation and that Ca^2+^ binding MICU2 increases the activity/function of the SDH/GPD2 metabolon independent of MCU and acute _m_Ca^2+^ uptake.

## Discussion

The evolution of Ca^2+^ sensing, signaling, and transport predates the transition from unicellular to multicellular life^59^. With the selection of phosphate as the energetic currency of life via ATP hydrolysis, the ability to export Ca^2+^ to maintain low intracellular [Ca^2+^] became advantageous to mediate sequestration of phosphate and avoid calcium-phosphate crystallization or precipitation. As life transitioned from unicellular to multicellular anywhere from ~700-million^60^ to ~2-billion^61^ years ago, ‘competition was replaced by cooperation’ and evolutionary pressure shifted to Ca^2+^ sensing and signaling^59^. The ability to transduce environmental Ca^2+^ as a proxy for the presence/absence of other organisms allowed for more efficient and effective resource utilization and sharing and survival of multicellular life^59^. When sustained evolutionary pressure on environmental Ca^2+^ sensing and signaling is considered in the context of endosymbiotic theory, one can easily postulate that organelles harbor sophisticated Ca^2+^ signal transduction machinery on the outer leaflet of the organellar membrane or envelope. Thus, Ca^2+^ sensing, signaling, and transport at and across organellar membranes are intrinsically tied to ATP production as these cellular processes have experienced equivalent evolutionary pressure.

Original biochemical studies performed and published in the 1970’s established two fundamental notions that sit at the foundation of the existing model of how Ca^2+^ regulates metabolism. First was the finding that mitochondria contained a channel capable of supporting inward rectifying Ca^2+^ current^62–64^. Second was the demonstration in reductionistic systems that critical metabolic enzymes located in the mitochondrial matrix, chiefly pyruvate dehydrogenase^5^, were sensitive to Ca^2+65^. These findings lend themselves to a model in which acute changes in _matrix_Ca^2+^ uptake and [_matrix_Ca^2+^] controls the activity of Ca^2+^ sensitive enzymes in the matrix. Over the last 15 years, the genetic identification and functional characterizations of various mitochondrial calcium handling components has reinvented the field and improved our understanding of this system in both physiology and disease. Even so, the mitochondrial and metabolism fields remain puzzled by discrepancies in the literature, namely that ablation of mitochondrial calcium uptake is phenotypically silent at baseline, while deletion of regulatory subunits (MICU1) or ablating the primary efflux pathway (NCLX) is lethal. These discrepancies in genetic manipulation of _matrix_Ca^2+^ uptake and efflux pathways can be explained in one of two ways.

The first possible explanation is that there is a secondary mode of uptake that is capable of compensating for loss of the principal uptake channel (mtCU), but not efflux (NCLX), pathways. Although other mitochondrial Ca^2+^ transporters do exist, namely the mitochondrial Ca^2+^/H^+^ antiporter Leucine zipper/EF‑hand‑containing transmembrane protein 1 (LETM1)^66^, these transports contribute minimally, if at all, to acute _matrix_Ca^2+^ uptake^67^. Further, although it has been suggested that mtCU-independent modes of _matrix_Ca^2+^ uptake are relevant to certain muscular pathophysiology^68^ this mechanism was proposed to be through altered permeability transition and cell death rather than metabolic perturbation and was only observed in the context of genetic deletion of *Mcu*. Given the acute timescale in which metabolic adaptation must occur and the near complete ablation of acute _matrix_Ca^2+^ uptake that occurs with loss or inhibition of mtCU function, it is highly unlikely that physiological Ca^2+^-mediated regulation of mitochondrial metabolism occurs within the matrix or that it is mediated by a mitochondrial calcium uptake pathway other than the mtCU.

The second possible explanation is that [_matrix_Ca^2+^] is not the master regulator of basal mitochondrial metabolism. Although initially perplexing, this notion is supported by the many studies that report mtCU function is dispensable to homeostasis^15,17,19,22,24–26^. Prior to the discovery of the uniporter’s genetic identity, Kirichok *et al*. demonstrated that the uniporter is largely inactive at resting _cytosolic_[Ca^2+^] (<100 nM) or during _cytosolic_[Ca^2+^] rise that occurs with stimulation (100–500 nM)^69^. In combination with other reports^70,71^, this suggests that the uniporter relies on being localized to high-Ca^2+^ microdomains, namely mitochondria associated membranes (MAMs), or becomes active during extremely high workload or Ca^2+^-overload (i.e., pathology). The uniporter’s relevance during Ca^2+^-overload pathology, injury, or stress is well characterized and universally accepted by leaders in the field. The mtCU initially functions to meet the energetic crisis but ultimately triggers permeability transition with prolonged stress^15,17^. This implies that the uniporter is primarily relevant during stress, injury, or insult. The notion that uniporter localization may affect channel conductance was partially addressed by our co-authors showing that the uniporter is basally housed within the cristae membrane and during stimulation shuttles to the inner boundary membrane in a MICU1-dependent manner^43^. Interestingly, the majority of MICU1 and MCU do not basally colocalize in the absence of stimulation, suggestive that MICU1 does not basally occlude the uniporter as has previously been suggested^34,35^. Further this baseline positioning of the uniporter within the cristae membrane, not directly at a Ca^2+^ microdomain capable of reaching [Ca^2+^] substantial enough to trigger its activation, has prompted further work suggesting that MICU1 regulates uniporter activity through remodeling of mitochondrial cristae architecture and mitochondrial substructure membrane potential gradients^72^. However, our previous finding MICU1 modulates mitochondrial architecture *independent* of the uniporter^42^ brings into question the primary function of MICU proteins. Even so, the view that [_matrix_Ca^2+^] is not the principal regulator of metabolism during physiology but becomes relevant during pathology resolves the discrepancies in the literature, however one essential question remains: *How then does calcium regulate metabolism in the absence of stress?*

We propose that the MICU interactome serves as a fundamental calcium registry at the inner mitochondria membrane and is the primary physiological Ca^2+^ signaling mechanism regulating cellular metabolism and energetics. In regulating localization, formation, and function of the GPD2/C-II metabolon we demonstrate proof of concept for the existence this system. We propose that upon introduction of pathological stress, the uniporter becomes relevant as a high-capacity Ca^2+^ transporter to increase ATP bioavailability in response to stress and with prolonged stress (Ca^2+^-overload), uniporter activation causes metabolic derangement, permeability transition, and cellular demise. These findings challenge the _matrix_Ca^2+^-centric dogma that bioenergetic Ca^2+^ signaling occurs exclusively in the mitochondrial matrix and support a new concept: that bioenergetic Ca^2+^-driven metabolon formation is the primary means by which mitochondria meet the energetic demand of cellular workload.

Although it has long been hypothesized that individual mitochondria within a single cell are functionally distinct, only recently was it causally shown that distinct subpopulations of mitochondria specialize in oxidative versus reductive biosynthesis^73^. One could easily hypothesize that sophisticated microenvironmental signal transduction pathways, such as the one we outline here, exist to activate unique metabolic programs within individual mitochondria allowing them to better serve their microenvironment.

In the current report, we discovered and outlined how MICU proteins are essential to bioenergetic mitochondrial Ca^2+^ signaling, independent of [_matrix_Ca^2+^]. Using a novel equimolar MICU expression platform, we demonstrate that MICU heterodimerization and stoichiometry are independent of _matrix_Ca^2+^ uptake and acutely sensitive to physiological Ca^2+^ signaling at discrete microdomains within the IMS. We utilized an IP/LC/MS approach to demonstrate unique MICU1/2 and MICU1/3 heterodimer interactomes and show that the MICU1/2 heterodimer binds to and determines coupling of GPD2 and SDH/C-II. SDH/C-II is unique as it functions as both an ETC complex and TCA cycle enzyme. Given the duality of SDH/C-II biology, one would expect distinct signaling mechanisms governing SDH/C-II flux coupling to the TCA cycle versus ETC. However, very little is known related to C-II regulation beyond the identification of SDH/C-II assembly factors (SDHAF1^74,75^, SDHAF2^76^, SDHAF3^77^, SDHAF4^78^). Given the energetic cost of complex assembly/disassembly and acute timescale that metabolic adaptation must occur, especially when considering the energetic responsiveness of oxidative phosphorylation, there is a substantial gap in knowledge with regards to SDH/C-II regulation. The identification of MICU2 as a regulator of SDH/C-II function not only underscores the validity of our model but is a substantial step forward in the understanding of SDH/C-II biology.

Similar to SDH/C-II, GPD2 functions in multiple metabolic pathways and represents a metabolic nexus. GPD2 converts glycerol-3-phosphate to dihydroxyacetone phosphate, reduces FAD^+^ to FADH_2,_ and, like SDH/C-II, feeds electrons to C-III in the form of reduced CoQ. GPD2 is essential to glycolysis, glycerophospholipid biosynthesis, and the glycerophosphate shuttle. Despite superficial differences in substrate, product, or cofactors–SDH/C-II and GPD2 are critical contributors to the CoQ pool, passing electrons to C-III independent of NADH and C-I. GPD2 itself serves as a critical source of electron leak^45,79^ and it has been suggested that GPD2-dependent ROS formation occurs at SDH via reverse mode electron transport^80^. One can envision that functional coupling of SDH/C-II and GPD2 would serve as an elegant system for controlling redox balance and oxidative phosphorylation.

The MICU2/GPD2/C-II metabolon that we have identified is a metabolic nexus, existing at the intersection of glycolysis, the TCA cycle, and the electron transport chain. Thus, we have characterized a calcium control point at the intersection of the three major metabolic pathways independent of acute _matrix_Ca^2+^ uptake.

## Methods:

### Cell culture:

All cells used in this study were cultured in Dulbecco’s Modified Eagle’s Medium (DMEM) with 4.5 g/L glucose, L-glutamine, and sodium pyruvate (Sigma Aldrich, cat #; D6429) supplemented with 10% fetal bovine serum (peak serum, cat #: PS-FB3) and 1% penicillin/streptomycin (Sigma-Aldrich, cat #: P0781–100ML). All cells used for this study were cultured at 37°C in the presence of 5% CO_2_. All knockout Neuro-2a (N2a) cell lines used for this study (*Mcu*^−/−^, *Micu1*^−/−^, *Micu2*^−/−^, *Mcu*^−/−^*Micu2*^−/−^) were generated according to the following procedure. Cells were transfected with plasmids expressing hCas9, 2 guide RNA’s antisense the target of interest, and a resistance gene to either hygromycin(Sigma Aldrich, cat #: H3274–1G), blasticidin (Invivogen, cat #: ant-bl-05), or neomycin/G418 (Fisher Scientific, cat #:10131035). Plasmids were transfected into cells using FugeneHD (Promega, cat #: E2311). 48 hours after transfection, media was replaced with media containing selection agent. After clearing all non-transfected cells, the transfected population was plated sparsely on 15 cm dishes and clonal populations were isolated using cloning cylinders (corning, cat #: 3166–6). Clonal populations were expanded and screened for successful deletion of the gene of interest using western blot. All gRNA and plasmid details are available in Materials.

### Western blotting:

Cells were collected and washed once with room temperature PBS. Subsequently cells were lysed in 1x RIPA (EMD Millipore, cat #: 20–188) supplemented with 1x protease inhibitor (Sigma, cat #:04693124001) and 1x phosphatase inhibitor (Roche, cat #: 4906837001) and incubated on ice for 30 minutes. Lysates were then spun down at 13,000 g for 20 minutes and supernatant collected for protein determination using pierce 660 nm protein assay reagent (Thermo Fisher Scientific, cat #: 22660) following manufacturer’s instructions. Protein lysates were then diluted to 1μg/μl, supplemented with 1x laemmli buffer containing 5% β-mercaptoethanol, and boiled for 5 minutes at 95°C. Samples were then electrophoresed on NuPAGE 4 to 12% bis-tris protein gels (Thermo Fisher, cat #: WG1402BOX) in SDS-MOPS running buffer (Bioworld, cat #: 10530007–2) or SDS-PAGE gels poured in-house using protogel (30%) (National diagnostics, cat #:EC-890) and protogel 4x resolving buffer (National diagnostics, cat #: EC-892) in 1x tris/glycine/SDS running buffer (BioRad, Cat #: 161–0772). Gels were transferred to PVDF membranes (EMD Milipore, cat #: IPFL00010) using the owl hep-3 semidry transfer system (ThermoFisher, cat #: HEP-3). Membranes were blocked for 1 hour at room temperature in blocking buffer (Rockland, cat #: MB-070) and incubated overnight at 4 degrees in primary antibody. Membranes were then washed 3x for 5 minutes in Tris-buffered saline containing 0.1% tween (Sigma Aldrich, cat#: P9416) and incubated for one hour in secondary antibody diluted 1:10,000 in TBS-T. Membranes were again washed 3x for 5 minutes in TBS-T and imaged on a Licor Odessey system.

### Fast protein liquid chromatography:

Fast protein liquid chromatography (FPLC) was performed as previously described^24,42^ with adaptation described below. Size exclusion chromatography was performed using an Akta Pure FPLC system equipped with a Superose 6 increase 10/300 GL column (Cytiva, cat #: 29–0915-96). Bio-Rad gel filtration standards (Bio-Rad, cat #: 1511901) were used to calibrate the machine and estimate molecular weights of each fraction. Cells were cultured on 15 cm cell culture dish (Celltreat, cat #: 229651), trypsinized, washed once with 1x PBS, and lysed using 1x RIPA supplemented with 1x protease and 1x phosphatase inhibitor. Lysates were fractioned at a flow rate of 0.5 ml/min and fractions were concentrated using Amicon Ultra centrifugal filters (EMD Millipore, cat #: UFC500396). Concentrated fractions were used for western blotting according to procedure described above. Quantifications were performed in Image Studio software version 5.2.5 (Licor).

### Mitochondrial isolation:

Mitochondrial isolation was performed as described previously^42^ with adaptations described below. Cells were trypsinized, washed once with PBS, and resuspended in 5x cell pellet volume of ice-cold mitochondrial isolation buffer (10 mM HEPES, 200mM mannitol, 70 mM sucrose, 1mM EGTA, pH 7.5). Suspensions were then homogenized with a dounce homogenizer (40 strokes) and centrifuged at 500 g for 10 min at 4°C to obtain the mitochondrial suspension. Mitochondria were then pelleted by centrifuging at 12,000g for 15 minutes at 4°C and washed 2x with 1 ml of ice-cold mitochondrial isolation buffer.

### Blue Native Page (BN-PAGE):

BN-PAGE samples were prepared using mitochondria isolated from cells as described above. Mitochondrial pellets were resuspended in 2% digitonin in 1x BN-PAGE sample buffer (ThermoFisher, cat #: BN2008) and incubated on ice for 30 min. Samples were then spun down at 20,000g for 20 min at 4°C. Supernatants were then collected, supplemented with 1 μl of G-250 per 10 μl of sample, and run on NativePAGE 4–16% Bis-Tris gels (ThermoFisher, cat #: BN1002BOX). Gels were transferred onto PVDF in 1x NuPage transfer buffer (ThermoFisher, cat #: NP00061) at 25V for 1 hour at 4°C. Membranes were then allowed to dry, unstained ladder marked (ThermoFisher, cat #: LC0725), and rehydrated in methanol. Membranes were then blocked in 5% BSA in TBS-T for 1 hour at room temperature before being incubated in the indicated primary antibody overnight at 4°C. Membranes were then washed 3x in TBS-T and incubated in the appropriate HRP-conjugated secondary antibody for 1 hour at room temperature. Membranes were washed 3x and developed in substrate for 5 minutes (ThermoFisher, cat #: 34580) and imaged using a Chemidoc Imaging System (Biorad).

### 2D gel electrophoresis:

Blue native page was performed as described above. Following electrophoresis, each lane of protein was cut out of the gel and soaked in 1% BME 1% SDS for 1 hour at room temperature. An SDS-PAGE resolving gel was poured as described above. In place of stacking gel, the BNPAGE gel strip in placed and secured using resolving gel and allowed to set. The second dimension of the gel was run, transferred to PVDF, and used for western blotting as described above.

### Measurements of mitochondrial calcium flux:

Measurement of mitochondrial Ca^2+^ flux was performed as previously described^81^ with slight modification. In brief, cells were washed with PBS, collected using 0.05% trypsin solution (Hyclone, cat: SH30236.01), and washed once in ice cold calcium-free Hank’s balanced salt solution (HBSS) (Sigma Aldrich, cat: H6648). An equal number of cells (4 million) were spun down and permeabilized in an intracellular-like medium [120 mM KCl, 10 mM NaCl, 1 mM KH2PO4, and 20 mM Hepes-tris (pH 7.2)] containing 5 mM succinate, 10 μM CgP-37157 (Enzo life sciences, cat: BML-CM119–0005) to block _matrix_Ca^2+^ efflux, and 3 μM Thapsigargin (Enzo Life Sciences, cat: BML-PE180–0005) to inhibit SERCA. 1 μM FuraFF was added to suspensions to monitor extramitochondrial Ca^2+^ and a multiwavelength excitation dual-wavelength emission fluorimeter (Delta RAM, PTI) was used to monitor fluorescence emission (340/380 nm emission, 535 nm excitation).

### Immunoprecipitation:

48–72 hours after transfection, cells were collected via trypsinization for immunoprecipitation. Cells were washed once using room temperature phosphate-buffered saline (PBS) and pellets resuspended in PBS containing 0.2 mM dithiobis(succinimidyl propionate) (DSP) (Thermo Scientific, cat #: A35393) and rotated at room temperature for 30 minutes. Pellets were then spun down and lysed using Pierce IP lysis buffer (Thermo Scientific, cat #: 87788) supplemented with 1x protease inhibitor (Sigma, cat #:04693124001) and 1x phosphatase inhibitor (Roche, cat #: 4906837001) for 30 minutes on ice. Lysates were then spun down and the supernatant used to proceed with IP protocol. Following protein quantification and normalization, Anti-HA (Thermo Fisher, cat #:88837), -Myc (Thermo fisher, cat #: 88842), or - FLAG (Sigma Aldrich, cat #: M8823) beads were added to input samples and rotated overnight at 4°C. Subsequently, beads were washed 3x with ice cold Tris-buffered Saline containing 0.05% tween, resuspended in pierce IP lysis buffer containing 1x laemmli buffer, and eluted by boiling for 5 minutes at 95C. IP eluate was then used for western blotting protocol as described above.

### Plasmid construction:

MICU1, MICU2, and MICU3 single, double, and triple expression plasmids and MICU1^EFmut^/MICU2^EFmut^/MICU3^EFmut^ were custom cloned by Vector Builder Inc. MICU1^EFmut^/MICU2/MICU3 was generated by cutting the MICU1 and MICU1^EFmut^ out of their respective plasmids using AscI (New England Biolabs, cat #:R0558S) and EcoNI (New England Biolabs, cat #: R0521S) and ligating the mutant gene fragment into the WT vector using T4 ligase (New England Biolabs, cat #: M0202S). MICU1/MICU2^EFmut^/MICU3 was generated by cutting MICU2 and MICU2^EFmut^ out of their respective plasmids using BamHI (New England Biolabs, cat #: R3136S) and NsiI (New England Biolabs, cat #: R0127S) and ligating the mutant fragment into the WT vector. MICU1/MICU2/MICU3^EFmut^ was generated by cutting MICU3 and MICU3^EFmut^ out of their respective vectors using BstEII (New England Biolabs, cat #: R3162S) and MfeI (New England Biolabs, cat #: R3589S) and ligating the mutant gene fragment into the WT vector. Ligation reactions were transformed into chemically competent cells (New England Biolabs, cat #: C2987I). Single clones were amplified and plasmid DNA was collected via miniprep (Macherey Nagel, cat #: 740588.25) and sequenced via next generation sequencing (Azenta Life Sciences, Plasmid EZ whole plasmid sequencing) to validate successful generation of desired product.

### Protein mass spectrometry sample preparation:

S-Trap micro columns were purchased from Protifi (Huntington, NY). Protein digestion in the S-Trap filter was performed following the manufacturer’s protocol. Briefly, equal of 2x SDS protein solubilization buffer (10% SDS, 100 mM triethylammonium bicarbonate, TEAB, pH 7.55) was added to equal volume of IP eluate. The proteins were reduced by addition of tris-(2-carboxyethyl)-phosphine (TCEP) to a final concentration of 5mM TCEP and incubating at RT for 30 min. Alkylation was carried out by adding chloracetamide (CAA) to a final concentration of 15 mM and incubating at RT for 30 min. Phosphoric acid was added to a final concentration of 2.5% followed by six volumes of binding buffer (90% methanol; 100 mM triethylammonium bicarbonate, TEAB; pH 7.1). After gentle mixing, the colloidal protein solution was loaded to an S-Trap columns, spun at 1500xg for 2 min, and the flow-through collected. The wells were washed 4x with binding buffer and sequencing-grade trypsin (Promega) in digestion buffer (100 mM TEAB) was added in the wells in 1:10 (w/w) ratio and digested at 47 °C for 3 h. Peptides were eluted using stepwise elution method with 50 mM TEAB, 0.2% formic acid in water, and 50% acetonitrile and 0.2% formic acid in water into new tubes. The elution was vacuum dried for further processing.

### Protein mass spectrometry sample processing:

Prior to LC-MS/MS analysis, dried peptides were reconstituted with 2% ACN, 0.1% FA and concentration was determined using a NanoDropTM spectrophometer (ThermoFisher). Samples were then analyzed by LC-MS/MS using a Proxeon EASY-nanoLC system (ThermoFisher) coupled to a Orbitrap Fusion Lumos Tribid mass spectrometer (Thermo Fisher Scientific). Peptides were separated using an analytical C18 Aurora column (75μm × 250 mm, 1.6 μm particles; IonOpticks) at a flow rate of 300 nL/min (60C) using a 75-min gradient: 2% to 6% B in 1 min, 6% to 23% B in 45 min, 23% to 34% B in 28 min, and 34% to 48% B in 1 min (A= FA 0.1%; B=80% ACN: 0.1% FA). The mass spectrometer was operated in positive data-dependent acquisition mode. MS1 spectra were measured in the Orbitrap in a mass-to-charge (m/z) of 375 – 1500 with a resolution of 60,000. Automatic gain control target was set to 4 × 10^5 with a maximum injection time of 50 ms. The instrument was set to run in top speed mode with 1-second cycles for the survey and the MS/MS scans. After a survey scan, the most abundant precursors (with charge state between +2 and +7) were isolated in the quadrupole with an isolation window of 0.7 m/z and fragmented with HCD at 30% normalized collision energy. Fragmented precursors were detected in the ion trap as rapid scan mode with automatic gain control target set to 1 × 10^4^ and a maximum injection time set at 35 ms. The dynamic exclusion was set to 20 seconds with a 10-ppm mass tolerance around the precursor.

### Protein mass Spectrometry Data analysis:

All mass spectra from were analyzed with MaxQuant software version 1.6.11.0. MS/MS spectra were searched against the Homo sapiens Uniprot protein sequence database (downloaded in Mar 2022), and GPM cRAP sequences (commonly known protein contaminants). Precursor mass tolerance was set to 20ppm and 4.5ppm for the first search where initial mass recalibration was completed and for the main search, respectively. Product ions were searched with a mass tolerance 0.5 Da. The maximum precursor ion charge state used for searching was 7. Carbamidomethylation of cysteine was searched as a fixed modification, while oxidation of methionine and acetylation of protein N-terminal were searched as variable modifications. Enzyme was set to trypsin in a specific mode and a maximum of two missed cleavages was allowed for searching. The target-decoy-based false discovery rate (FDR) filter for spectrum and protein identification was set to 1%.

### Complex II activity assay:

Complex II activity assay was performed on a Seahorse XF pro Analyzer as previously described^82^ with slight adaptation. Cells were plated (30k/well) in 96-well microplates (Agilent, cat #: 103774–100) the day before the experiment. The day of the experiment, cells were washed 2x with Mannitol Sucrose buffer (MSA) (70 mM sucrose, 220 mM mannitol, 10 mM KH2PO4, 5 mM MgCl2, 2 mM HEPES, 1 mM EGTA, pH 7.2) containing 4% fatty acid-free BSA (Sigma Aldrich, cat #: A7030–10G) (MSA-BSA) and loaded into the machine. Following injection 1, each well contained 1 nM PMP (Agilent, cat #: 102504100 and United States Biological, cat#: 370743), 10 mM succinate (Sigma Aldrich, cat #: S3674–100G), 1 mM ADP (Sigma Aldrich, cat #: A5285–1G), 1μM Rotenone (Sigma Aldrich, cat #: R8875). Following injection 2 each well contained 1 ug/ml oligomycin (Sigma Aldrich, cat #: O4876) and following injection 3 each well contained 20 μM Antimycin A (Sigma, cat #: A8674). Data analysis was performed in Seahorse Wave software (Agilent).

### Liquid chromatography-high resolution metabolite mass spectrometry:

Metabolites and stable isotope incorporation were measured by liquid chromatography-high resolution mass spectrometry adapted from previously published approaches^83^. Samples were quenched with 1 mL pre-chilled −80°C 80/20 methanol (Fisher Scientific: A456–500):water (Fisher Scientific: W64) (v/v). After vortexing for 1 min samples were returned to −80°C 30 min, centrifuged 17,000 x g 10 min at 4°C, and supernatant was transferred to a 96-well plate and evaporated to dryness under nitrogen gas. Samples were reconstituted in 50 μL then 2 μL of the sample kept at 4 °C was injected from an autosampler onto a 25 °C ZIC-pHILIC 150 × 2.1 mm 5 μm particle size column (EMD Millipore: 1504610001) with a ZIC-pHILIC 20 × 2.1 guard column (EMD Millipore: 1504380001) in a Vanquish Duo UHPLC System (Thermo Fisher Scientific). Chromatography conditions were as follows: buffer A was acetonitrile (Fisher Scientific: A9554); buffer B was 20 mM ammonium carbonate (Thermo Scientific: 401130250), 0.1% (v/v) ammonium hydroxide (Fisher Scientific: A955–4) in water without pH adjustment, with a gradient of 0.5 min at 20% A then a linear gradient from 20% to 80% B; 20–20.5 min: from 80% to 20% B; 20.5–28 min: hold at 20% B at a 0.150 mL/min flow rate. Column eluate was introduced to a Q Exactive Plus with a HESI II probe operating in polarity switching mode with full scans from 70–1000 m/z with an insource fragmentation energy of 1. Instruments were controlled via XCalibur 4.1 and data was analyzed on Tracefinder 5.1 using a 5ppm window from the predominant M-H negative ion.

### Glycerol-3-phosphate activity assays:

GPD2 activity assays were performed as described previously^57,58^. In brief, mitochondria membranes were obtained using a previously described freeze-thaw technique and treated with 10 mM glycerol-3-phosphate (Sigma Aldrich, cat#: 94124) to drive GPD2 activity. GPD2 activity was quantified by reduction of 2,6-dichlorophenol-indophenol (DCPIP) (Sigma Aldrich, cat#: D1878) measured via absorbance at 600 nm in the presence of antimycin A and rotenone. GPD2 + CIII activity was measured via reduction of cytochrome c indicated by absorbance at 550 nm in the presence of rotenone. All absorbance measurements were performed at 37°C on a Tecan Infinite M1000 Pro plate reader.

### Structured Illumination Microscopy (SIM):

The SIM setup used is composed of a 405, 488, 515, 532, and a 561 nm excitation laser introduced at the back focal plane inside the SIM box with a multimodal optical fiber. For super-resolution, a CFI SR Apochromat TIRF ×100-oil (NA 1.49) objective was mounted on a Nikon-Structured Illumination Microscopy (N-SIM^®^, Nikon, Austria) System with standard wide field and SIM filter sets and equipped with two Andor iXon3^®^ EMCCD cameras mounted to a Two Camera Imaging Adapter (Nikon Austria, Vienna, Austria). At the bottom port, a third CCD camera (CoolSNAP HQ2, Photometrics, Tucson, USA) is mounted for wide-field imaging. For calibration and reconstruction of SIM images, the Nikon software (NIS-Elements AR 4.51.00 64-bit, Nikon, Austria) was used. Reconstruction was permanently performed with the same robust setting to avoid artifact generation and ensures reproducibility with a small loss of resolution of 10% compared to the most sensitive and rigorous reconstruction settings. Microscopy setup adjustments were done as described elsewhere^43^.

### Förster resonance energy transfer (FRET) imaging:

The setup used is composed of a celesta light engine (Lumencor, USA, Beaverton) with 405, 446, 477, 520, 546, 638, and 749 nm excitation lasers introduced at the Xlight V3 spinning disk unit (Crestoptics, Italy, Rom) with a multimodal optical fiber. For widefield FRET measurements a pE-800 (CoolLED, UK, Andover) LED light source with 365, 400, 435, 470, 500, 550, 580, 635, and 740 nm LEDs is introduced at the back focal plane. A plan apo λD 100X-oil (NA 1.45) objective is mounted on a Nikon ECLIPSE Ti2 (Nikon, Austria) System with standard wide field and confocal filter sets and equipped with two back-illuminated Kinetix Scientific CMOS cameras (TELEDYNE PHOTOMETRICS, USA, Tucson) mounted to the Xlight V3 spinning disk unit. The Nikon software (NIS-Elements AR 5.42.06 (Build 1821) LO 64bit, Nikon, Austria) was used for microscope control and image acquisition. Cells were seeded on glass clover slips and transfected with indicated FRET pairs. Time lapse experiments were conducted with a sampling rate of 0.5 Hz with alternating illuminations of 435 nm and 500 nm. For 435 nm illumination both CFP (F_CFP_) and FRET (F_FRET_) fluorescence intensities were recorded, for 500 nm illumination only YFP (F_YFP_) fluorescence intensity was measured. F_CFP_, F_YFP_, and F_FRET_ were background corrected using a background region of interest and bleaching corrected using an extrapolation of an exponential decay function retrieved from basal measurements of each cell. Afterwards, intermolecular FRET was calculated as:

$$\frac{\frac{F_{FRET}}{F_{YFP}}}{F_{CFP}}=FRET_{efficiency}$$

### Inner boundary membrane (IBM) association index:

The IBM association index was calculated as described elsewhere^43^. In short, images were subjected to background subtraction (Mosaic Suite, background subtractor, NIH) with a sliding rectangle diameter of 50 pixels. The reference channel (MTG, TMRE, MICU2-mCherry, MICU1-EGFP) was Otsu auto thresholded and further dilated and eroded in two independent subsets. Pixel-wise subtraction of the erosion reference of the dilated reference image yields a hollow structure, used as a mask to measure the mean intensity in the mitochondrial periphery or IBM-related area in the object channel. The erosion reference served as a mask to measure the bulk or cristae mean fluorescence intensity. The ratio of IBM/CM mean intensity is a value to estimate changes in the object label distribution inside a mitochondrion, which is referred to as the IBM association index. The higher the ratio value the higher the distribution of protein labels in the IBM. For image analysis the freeware program ImageJ was used.

### Generation and validation of MICU1-HA-MCU-V5 knock-in mouse:

gRNA’s were designed targeting the C-terminus of the Mcu and Micu1 alleles containing the V5 and HA epitopes, respectively. (MCU gRNA: ggatcttaagagactgagagacccattacaagtacacctgcccctccgacagatTggTgaGaaggaaggttctggatccggttctggaagtggatccggtaagcctatccctaaccctctcctcggtctcgattctacgtgatccgagatgaccgtgaatcccggcagagagtgcgcctgtttgtaactcacgc, MICU1 gRNA: ccatgtggaaatgtgcccaagaaactgcctgggactttgctctgcccaaaggttctggatccggttctggaagtggatcctacccatacgatgttccggattacgcttagtattcccacctcctgcaccttagcaccctgcaagccctggagtggcccttcatgc). Cas9 protein and gRNA’s were injected into C57BL6/J ova which were then implanted into female mice. Potential pups were screened for successful knock-in of the epitope tags using genotyping primers flanking the desired sites of insertion. All animals generated for this study were housed and bred using protocols approved by Temple Universities IACUC in accordance with AAALAC.

**Table:** 

Genotyping primers	Sequence (5’→3’)
MICU1-HA-F	ggagctgagcaacaaggagt
MICU1-HA-R	gcagaacactgatggggtca
MCU-V5-F	ggcaggtctttgctgagact
MCU-V5-R	cctccacctgccatgcttta

### Statistical analysis:

Results are presented as mean ± std error. Graphpad Prism 10 was used for all statistical analysis and data visualization. Comparisons made between 2 groups were made using a student’s unpaired two-tailed t-test. Comparisons made between 3 or more groups were done so using a one- or two-way ANOVA test with Tukey or Šídák’s multiple comparison test. P values <0.05 are indicated as significant.

## Materials:

**Table:** 

Plasmid	Source	Cat #
mMicu2 crisprCas9 targeting plasmid	Vectorbuilder inc.	VB201021–1516tex
mMCU crisprCas9 targeting plasmid	Vectorbuilder inc.	VB230919–1381mhs
Vector control	Vectorbuilder inc.	VB900129–0604upq
MICU1-HA	Vectorbuilder inc.	VB220824–1339ncd
MICU2-Myc	Vectorbuilder inc.	VB220824–1340hdv
MICU3-FLAG	Vectorbuilder inc.	VB220824–1343bfc
MICU1-HA-T2a-MICU3-FLAG	Vectorbuilder inc.	VB220824–1323hqq
MICU1-HA-T2a-MICU2-Myc	Vectorbuilder inc.	VB220824–1324hwb
MICU2-Myc-T2a-MICU3-FLAG	Vectorbuilder inc.	VB220824–1326gxq
MICU1-HA-T2a-MICU2-Myc-MICU3FLAG	Vectorbuilder inc.	VB220824–1320yrw
MICU1^EFmut^-HA-T2a-MICU2^EFmut^-MycMICU3^EFmut^-FLAG	Vectorbuilder inc.	VB231114–1153sbf
MICU1-EGFP	Vectorbuilder inc.	VB230908–1285guw
MICU2-mCherry	Vectorbuilder inc.	VB230908–1228qwa
MICU2-CFP	This study	
MICU2^EFmut^-Myc	This study	
MICU1-YFP	Waldeck-Weiermair, M. et al.^84^	

Antibodies			
Antibody	manufacturer	Manufacturer catalog #	RRID
Rat anti-HA	Roche	11867423001	AB_390918
Rabbit Anti-Myc	abcam	ab9106	AB_307014
Rat Anti-FLAG	Biolegend	637301	AB_1134266
Rabbit Anti-MICU1	Novus Biologicals	NBP1–86663	AB_11021790
Rabbit Anti-MICU2	Bethyl	A300-BL19212	
Rabbit Anti-MICU3	Sigma Aldrich	HPA024771–100UL	AB_1848022
Rabbit Anti-MCU	Sigma Aldrich	HPA016480	AB_2071893
Mouse Anti-ATP5a	abcam	ab14748	AB_301447
Mouse Anti-SDHA	abcam	ab14715	AB_301433
Mouse Anti-SDHB	abcam	ab14714	AB_301432
Rabbit Anti-SDHC	abcam	ab155999	AB_2810989
Rabbit Anti-SDHD	abcam	ab189945	AB_3665949
Rabbit Anti-GPD2	Proteintech	17219–1-AP	AB_2112476
Goat Anti-Rat 680 nm secondary	Licor	925–68076	AB_2814913
Goat Anti-Mouse 680 nm secondary	Licor	925–68070	AB_2651128
Goat Anti-Rat 800 nm secondary	Licor	926–32219	AB_1850025
Goat Anti-Mouse 800 nm secondary	Licor	926–32210	AB_621842
Goat Anti-Rabbit 680 nm secondary	Licor	926–68071	AB_10956166
Goat Anti-Rabbit 800 nm secondary	Licor	925–32211	AB_2651127
Goat anti-Mouse HRP secondary	ThermoFisher Scientific	31430	AB_228307

## Supplementary Material

Supplement 1

## Figures and Tables

**Figure 7.**
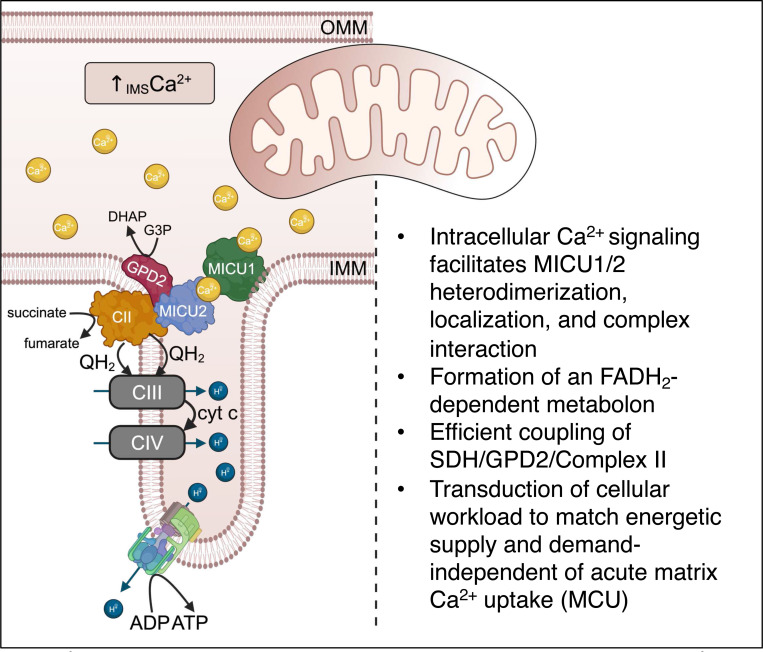
Ca^2+^-dependent metabolon formation couples SDH/GPD2 metabolism. Ca^2+^- sensing proteins MICU1 and MICU2 heterodimerize at the inner boundary membrane in response to physiological stimulation increasing [Ca^2+^] in the inter membrane space. The MICU1/2 heterodimer binds to and regulates the activity of the FADH_2_-dependent mitochondrial metabolon of SDH/Complex II (C-II) and glycerol-3-phosphate dehydrogenase (GPD2).

## Data Availability

All data are available in the main text or the supplementary materials. Any materials request can be directed to the corresponding author. Proteomics data associated with this manuscript has been deposited on the MassIVE proteomics database with the identifier MSV000096812 or on proteomeXchange under the identifier PXD059606.
