# Supplementary material for: MICU proteins facilitate Ca2+-dependent mitochondrial metabolon formation to regulate cellular energetics - independent of MCU
Source: Res Sq. 2025 Jun 26:rs.3.rs-6346822. Preprint. [Version 1] doi: 10.21203/rs.3.rs-6346822/v1 (PMC12270237; doi:10.21203/rs.3.rs-6346822/v1)
Supplement: Supplement 1 [file NIHPPRS6346822v1-supplement-1.pdf]

Supplemental Figure 1

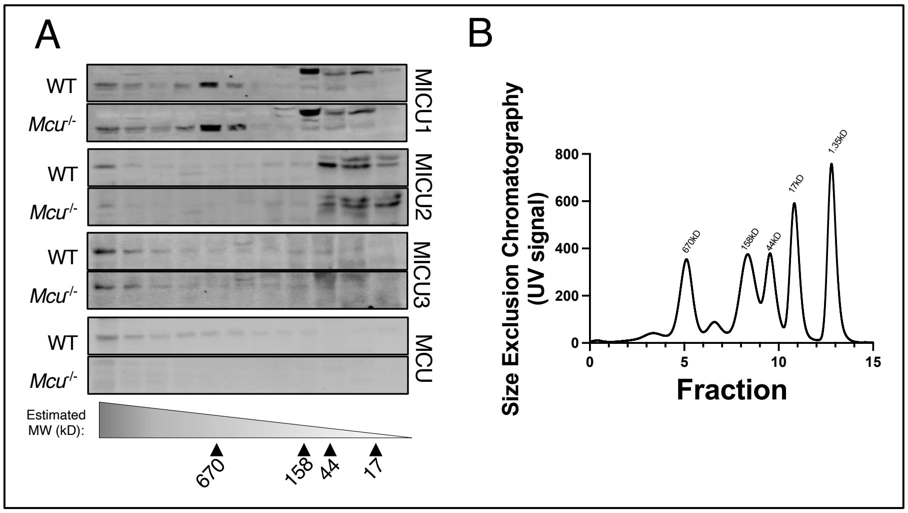

585 **Supplemental Figure 1. Size exclusion chromatography in WT and *Mcu*$^{-/-}$ N2a cells A.** Western blots corresponding to Fig. 1E **B.** Standards run to estimate molecular weights of proteins collected in during size exclusion chromatography.

Supplemental Figure 2

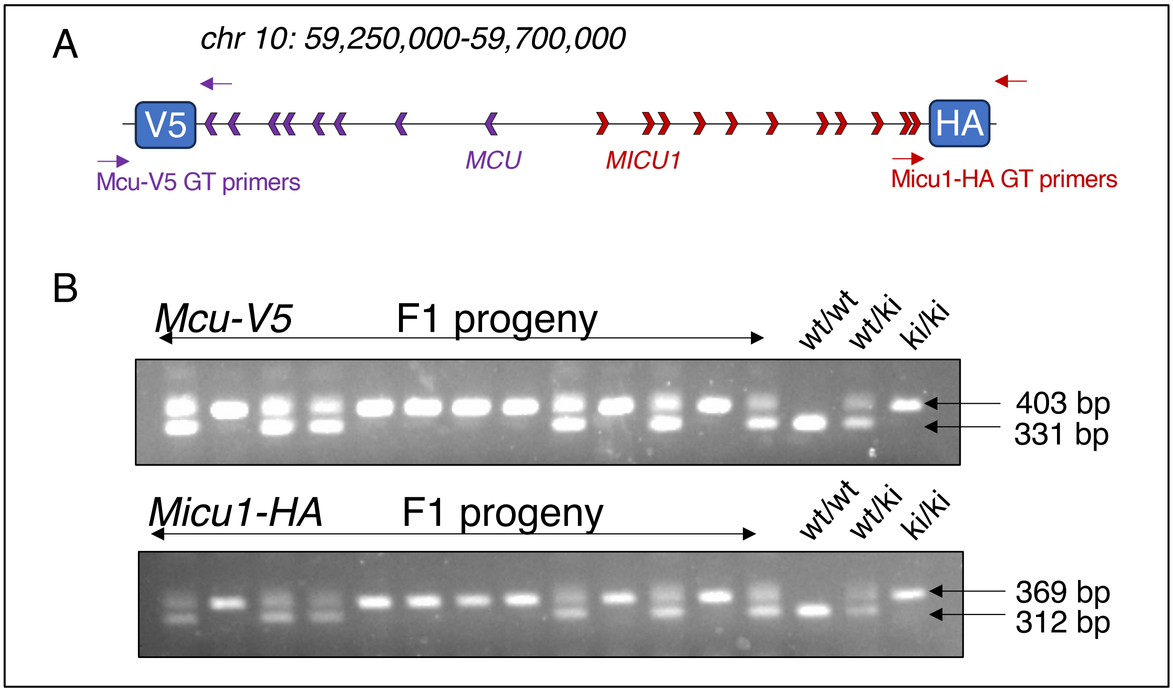

Supplemental Figure 2. Validation of MICU1-HA-MCU-V5 knock-in mouse. A. Schematic for integration of HA and V5 tag into the loci encoding the C-terminus of MICU1 and MCU protein, respectively, and genotyping primers used for validation. **B.** Genotyping of F1 generation demonstrating successful integration of HA and V5 tags into the endogenous gene loci of Micu1 and Mcu, respectively, on the same chromosome.

Supplemental Figure 3

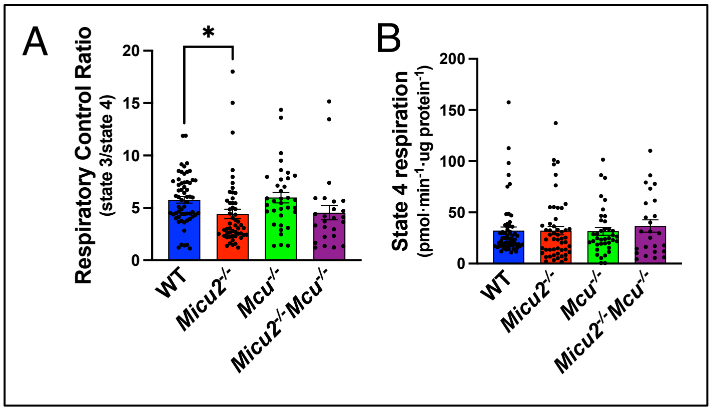

Supplemental Figure 3. Quantification of Complex II activity assay in WT, *Micu2*$^{-/-}$, *Mcu*$^{-/-}$, and *Micu2*$^{-/-}$*Mcu*$^{-/-}$ cells. A, B. Respiratory control ratio and state 4 respiration related to figure 6J. One-way ANOVA with Tukey's multiple comparison test.
